# Etiology of Severe Acute Watery Diarrhea in Children in the Global Rotavirus Surveillance Network Using Quantitative Polymerase Chain Reaction

**DOI:** 10.1093/infdis/jix294

**Published:** 2017-06-21

**Authors:** Darwin J Operario, James A Platts-Mills, Sandrama Nadan, Nicola Page, Mapaseka Seheri, Jeffrey Mphahlele, Ira Praharaj, Gagandeep Kang, Irene T Araujo, Jose Paulo G Leite, Daniel Cowley, Sarah Thomas, Carl D Kirkwood, Francis Dennis, George Armah, Jason M Mwenda, Pushpa Ranjan Wijesinghe, Gloria Rey, Varja Grabovac, Chipo Berejena, Chibumbya J Simwaka, Jeannine Uwimana, Jeevan B Sherchand, Hlaing Myat Thu, Geethani Galagoda, Isidore J O Bonkoungou, Sheriffo Jagne, Enyonam Tsolenyanu, Amadou Diop, Christabel Enweronu-Laryea, Sam-Aliyah Borbor, Jie Liu, Timothy McMurry, Benjamin Lopman, Umesh Parashar, John Gentsch, A Duncan Steele, Adam Cohen, Fatima Serhan, Eric R Houpt

**Affiliations:** 1 University of Virginia, Charlottesville;; 2 National Institute for Communicable Diseases, Johannesburg, and; 3 South African Medical Research Council/Diarrhoeal Pathogens Research Unit, Department of Virology, Sefako Makgatho Health Sciences University, Pretoria, South Africa;; 4 Christian Medical College, Vellore, India;; 5 Instituto Oswaldo Cruz/Fiocruz, Rio de Janeiro, Brazil;; 6 Murdoch Childrens Research Institute, Melbourne, Australia;; 7 Noguchi Memorial Institute for Medical Research, Accra, Ghana;; 8 World Health Organization (WHO) Regional Office for Africa, Brazzaville, Republic of the Congo;; 9 WHO Regional Office for South-East Asia, New Delhi, India;; 10 WHO Regional Office for the Americas, District of Columbia;; 11 WHO Regional Office for the Western Pacific, Manila, the Philippines;; 12 University of Zimbabwe, Harare;; 13 University Teaching Hospital, Lusaka, Zambia;; 14 Ministry of Health, Kigali, Rwanda;; 15 Tribhuvan University, Kathmandu, Nepal;; 16 Department of Medical Research, Yangon, Myanmar;; 17 Medical Research Institute, Colombo, Sri Lanka;; 18 Laboratoire National de Santé Publique, Ouagadougou, Burkina Faso;; 19 National Public Health Laboratories, Fajara, The Gambia;; 20 Sylvanus Olympio Teaching Hospital, Lomé, Togo;; 21 Albert Royer National Paediatric Hospital Laboratory, Dakar, Senegal;; 22 University of Ghana Medical School, Accra;; 23 University of Sierra Leone, Freetown;; 24 Emory University, and; 25 Centers for Disease Control and Prevention, Atlanta, Georgia;; 26 Bill & Melinda Gates Foundation, Seattle, Washington; and; 27 World Health Organization, Geneva, Switzerland

**Keywords:** diarrhea, PCR, rotavirus, surveillance

## Abstract

**Background:**

The etiology of acute watery diarrhea remains poorly characterized, particularly after rotavirus vaccine introduction.

**Methods:**

We performed quantitative polymerase chain reaction for multiple enteropathogens on 878 acute watery diarrheal stools sampled from 14643 episodes captured by surveillance of children <5 years of age during 2013–2014 from 16 countries. We used previously developed models of the association between pathogen quantity and diarrhea to calculate pathogen-specific weighted attributable fractions (AFs).

**Results:**

Rotavirus remained the leading etiology (overall weighted AF, 40.3% [95% confidence interval {CI}, 37.6%–44.3%]), though the AF was substantially lower in the Americas (AF, 12.2 [95% CI, 8.9–15.6]), based on samples from a country with universal rotavirus vaccination. Norovirus GII (AF, 6.2 [95% CI, 2.8–9.2]), *Cryptosporidium* (AF, 5.8 [95% CI, 4.0–7.6]), *Shigella* (AF, 4.7 [95% CI, 2.8–6.9]), heat-stable enterotoxin-producing *Escherichia coli* (ST-ETEC) (AF, 4.2 [95% CI, 2.0–6.1]), and adenovirus 40/41 (AF, 4.2 [95% CI, 2.9–5.5]) were also important. In the Africa Region, the rotavirus AF declined from 54.8% (95% CI, 48.3%–61.5%) in rotavirus vaccine age-ineligible children to 20.0% (95% CI, 12.4%–30.4%) in age-eligible children.

**Conclusions:**

Rotavirus remained the leading etiology of acute watery diarrhea despite a clear impact of rotavirus vaccine introduction. Norovirus GII, *Cryptosporidium*, *Shigella*, ST-ETEC, and adenovirus 40/41 were also important. Prospective surveillance can help identify priorities for further reducing the burden of diarrhea.

## Summary

We calculated pathogen-specific attributable fractions of acute watery diarrhea in children from 16 countries using quantitative polymerase chain reaction testing for a broad range of enteropathogens. Rotavirus remained the leading etiology, despite a clear impact of rotavirus vaccine introduction.

Diarrheal disease remains a leading public health burden in children <5 years of age in low-resource settings [1, 2], with rotavirus identified as the most common cause [1, 3]. As the global introduction of rotavirus vaccine (RV) continues, an accurate description of the etiology of diarrhea in these settings is needed to advance local and global preventive and treatment efforts. The recent application of quantitative polymerase chain reaction (qPCR) for detection of enteropathogens to studies of diarrhea has improved our understanding of diarrheal etiology [1, 4]. For example, a qPCR reanalysis of the Global Enteric Multicenter Study (GEMS) showed that the burdens of *Shigella*, heat-stable enterotoxin-producing *Escherichia coli* (ST-ETEC), and enteric adenoviruses 40 and 41 were substantially underestimated in 7 countries in Africa and Asia [4, 5]. However, this diagnostic approach has not been applied to a large number of countries or sites.

The World Health Organization (WHO)–coordinated Global Rotavirus Surveillance Network (GRSN) systematically collects and tests stool specimens collected from children hospitalized with acute watery diarrhea from 178 sentinel surveillance sites in approximately 60 countries representing all 6 WHO regions. The network was established in 2008 to provide rotavirus burden information to member countries, measure vaccine impact, and monitor strain epidemiology in a standardized manner across regions [6]. Specimen testing is carried out by the Global Rotavirus Laboratory Network whose components include reference laboratories at the sentinel hospital, national, regional, and global levels [7]. In this study, we leveraged these existing networks by equipping and training regional laboratories to perform qPCR using TaqMan Array Cards on archived diarrheal stool specimens. Using these specimens, we show the diversity and distribution of the etiology of acute watery diarrhea across 4 WHO regions and examine the etiology of diarrhea in children <5 years of age during the ongoing global introduction of RV by calculating the attributable fraction (AF) of diarrhea for each pathogen.

## METHODS

### Surveillance Design and Participants

Diarrheal episodes included in this analysis came from 4 WHO regions: the African Region, the Region for the Americas, the South-East Asia Region, and the Western Pacific Region. The GRSN enrolled children <5 years of age presenting with diarrhea of <14 days’ duration [6]. Bloody diarrhea was excluded, and only children admitted to the hospital were enrolled, except in Brazil where children were either hospitalized or outpatients. A stool sample was collected from all enrolled children and tested for rotavirus by enzyme immunoassay (EIA) using the Oxoid ProSpecT Rotavirus test (Oxoid, Cambridge, United Kingdom) or the Meridian Premier Rotaclone test (Cincinnati, Ohio).

### Stool Selection and Testing by qPCR

Participating laboratories that performed nucleic acid extraction and testing included the Noguchi Memorial Institute for Medical Research, Accra, Ghana; National Institute for Communicable Diseases, Johannesburg, South Africa; Christian Medical College, Vellore, India; Murdoch Childrens Research Institute, Melbourne, Australia; and Fiocruz, Rio de Janeiro, Brazil. These reference laboratories routinely receive specimens from countries for quality control and rotavirus genotyping [6]. Guidance was given that samples for molecular testing should be randomly selected from specimens shared by surveillance sites across calendar years 2013 and 2014, with a suggested goal of sampling 50% rotavirus EIA-positive and 50% EIA-negative samples, to allow ascertainment of other etiologies and evaluation of rotavirus genotypes. Nucleic acid was extracted with the QIAamp Fast DNA Stool mini kit (Qiagen, Hilden, Germany) using a modified protocol that included bead beating [8]. Two external controls, MS2 bacteriophage and phocine herpes virus, were added to samples during nucleic acid extraction to monitor extraction and amplification efficiencies. Extraction blanks and no-template controls were included to monitor contamination. The qPCR testing was performed using a custom TaqMan Array Card, which provided simultaneous qPCR for a broad range of enteropathogens [9]. The performance and interlaboratory reproducibility of the TaqMan Array Card method has been described previously [9]. All assays on the card were identical to those used previously in the qPCR reanalysis of the GEMS study [4]. Valid results required proper functioning of MS2 and phocine herpes virus controls and excluded data flagged by the data acquisition/analysis software because of excessive noise in the fluorescence signal of the ROX reference dye. For quality assurance, all testing sites underwent a 1-week training session (by D. J. O.) and used a common set of standard operating procedures. For quality control, negative controls were carried through extraction, spiked internal controls were included in each sample, and a common scheme for data analysis was used, as well as common positive controls. To guard against low-level detection of pathogens in extraction blanks, a quantification cycle value of 30 was used as the limit of detection for all pathogens.

### Analysis

This study involved retrospective testing of diarrheal specimens. Because it has been recognized that asymptomatic infection with enteropathogens is common in these settings, particularly when using molecular detection methods, we calculated the population AF for each pathogen, where the AF can be interpreted as the proportion of disease cases that would not occur in the absence of the risk factor. To calculate AFs, we first calculated the strength of association between the quantity of pathogen detected and diarrhea using models developed from a qPCR reanalysis of the multisite GEMS case-control study of diarrhea [4, 5]. In brief, using qPCR data from 4077 cases of moderate-to-severe acute watery diarrhea (ie, excluding dysentery) and age-, sex-, and village-matched controls from the GEMS study, we fit a multivariable conditional logistic regression model [4] to describe the association between pathogen quantity and diarrhea while adjusting for the presence of other pathogens. These pathogen- and quantity-specific odds ratios were then used to calculate AFs for the current dataset based on the quantity of each pathogen detected in each stool. Specifically, because of the nonrandom selection of diarrheal episodes for qPCR testing in the current study, we calculated weighted population AFs by summing pathogen attributions across each of *j* cases with valid qPCR results in the current study, namely $AF_{i}\;=\;1-\sum_{1}^{j}\left(W_{i}/OR_{i}\right)/\sum_{1}^{j}\left(W_{i}\right)$ where $W_{i}$ is the inverse of the predicted probability of selection for qPCR testing derived from a logistic regression model using all episodes identified via surveillance, with age, month of year, EIA result, and country as well as an interaction between EIA result and country as the covariates and *OR*_*i*_ is the pathogen-specific odds ratios from the GEMS models [11]. Weights beyond the 99th percentile were trimmed to the 99th percentile. The same approach was applied to calculate the weighted prevalence of each pathogen, and the original GEMS models were used to calculate rotavirus AF estimates by EIA, but with the EIA result removed from the weighting. This inverse probability weighting strategy corrects for the under- or overrepresentation of subsets of the surveillance data by age, calendar month, country, and EIA result.

For AF estimates, a 2-step procedure was used to estimate error. To account for surveillance site sampling variability, data was bootstrapped 100 times. Each bootstrap iteration was analyzed using the models fit for each of the 7 GEMS sites after randomly perturbing the GEMS model coefficients in accordance with their sampling variance-covariance; this random perturbation of coefficients was performed to account for sampling variability in the GEMS model fit. The 95% confidence intervals were derived from the 2.5th and 97.5th quantiles of the AF distribution, and the point estimate of the AF was calculated using the original dataset and model coefficients. The one-sample proportion test was used to calculate 95% confidence intervals (CIs) for the proportion of samples positive by rotavirus EIA. Children were considered age eligible to receive RV if they were at least 2 months of age at enrollment and were born no more than 2 months prior to the country’s month of RV introduction.

Because individual vaccine histories were not available, we estimated the effectiveness of RV introduction by fitting a logistic regression model with the EIA test result as the response and age eligibility, age using a natural spline with knots at 6, 12, and 18 months, sex, site, and season via the terms $sin\left(\frac{2m\pi }{12}\right)+cos\left(\frac{2m\pi }{12}\right)+\;sin\left(\frac{4m\pi }{12}\right)+cos\left(\frac{4m\pi }{12}\right)$, where *m* is the month of the year. We also included an interaction between the seasonal terms and site, given possible variation in seasonality between sites [11]. Age and season terms were chosen based on model fit as assessed by the Akaike information criterion. The effectiveness of RV introduction was then calculated as (1 – OR) × 100, where OR was the exponential of the coefficient for age eligibility. All analyses were performed using R version 3.3.1 software.

## RESULTS

**Figure 1.**
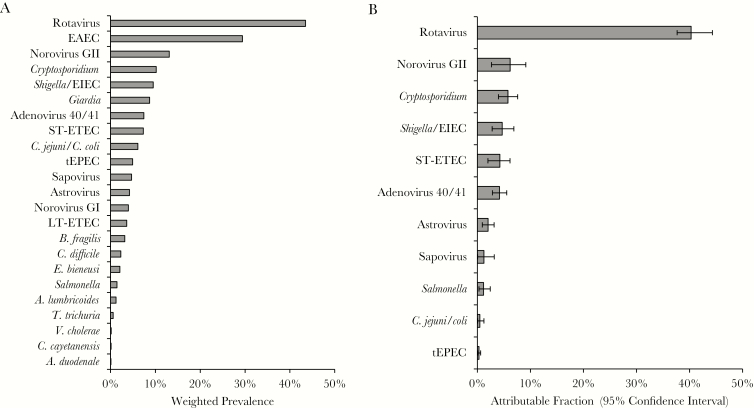
Weighted prevalence of enteropathogens tested by quantitative polymerase chain reaction (*A*) and pathogen-specific burdens of diarrhea across all World Health Organization regions (*B*). The weighted prevalence is shown for all pathogens with at least 1 detection (*A*), while overall weighted attributable fractions are shown for all pathogens for which the 95% confidence interval did not include 0 (*B*). Abbreviations: EAEC, enteroaggregative *Escherichia coli*, EIEC, enteroinvasive *Escherichia coli*; LT-ETEC, heat-labile enterotoxin-producing *Escherichia coli*; ST-ETEC, heat-stable enterotoxin-producing *Escherichia coli*; tEPEC, typical enteropathogenic *Escherichia coli.*

A total of 15674 episodes of acute watery diarrhea from children <5 years of age were enrolled in 2013–2014 across 16 countries from 4 WHO regions. A corresponding stool EIA result was not available for 993 episodes, and the child’s age was not available for 38, leaving 14643 episodes (Table 1). Of these, 878 (6.0%) were tested by qPCR, and valid qPCR results were available for all pathogens in 840 of 878 (95.7%). There was at least 1 detection by qPCR for 23 of the 30 interrogated enteropathogens (Figure 1A). Rotavirus was the most commonly detected enteropathogen, followed by enteroaggregative *E. coli*, norovirus GII, *Cryptosporidium*, *Shigella*/enteroinvasive *E. coli* (EIEC), and *Giardia*. Aside from *Giardia* and *Cryptosporidium*, detection of intestinal parasites was uncommon.

**Table 1.** Diarrhea Captured by Surveillance in 2013–2014 and Subset Tested by Quantitative Polymerase Chain Reaction

WHO Region	Country	All Diarrhea Captured in 2013–2014			Subset of Diarrhea With Valid qPCR Results		
		No.	Median Age, mo (IQR)	Rotavirus EIA Positive, No. (%)	No. (%)	Median Age, mo (IQR)	Rotavirus EIA Positive, No. (%)
Africa Region	Benin	157	9 (6–12)	64 (40.8)	10 (6.4)	8 (7–13)	5 (50.0)
	Burkina Faso	824	9 (6–14)	385 (46.7)	27 (3.3)	10 (5–14)	18 (66.7)
	Ghana	730	11 (6–18)	227 (31.1)	11 (1.5)	8 (6–13)	9 (81.8)
	The Gambia	260	14 (9–23)	59 (22.7)	20 (7.7)	10 (8–17)	15 (75.0)
	Mauritius	643	18 (11–30)	352 (54.7)	35 (5.4)	22 (13–30)	26 (74.3)
	Rwanda	1627	12 (9–18)	341 (21.0)	42 (2.6)	13 (9–17)	25 (59.5)
	South Africa	1337	9 (5–15)	351 (26.3)	52 (3.9)	10 (6–16)	10 (19.2)
	Senegal	234	10 (4–19)	87 (37.2)	16 (6.8)	8 (4–14)	15 (93.8)
	Sierra Leone	351	9 (6–13)	154 (43.9)	10 (2.8)	8 (5–12)	5 (50.0)
	Togo	396	10 (7–14)	231 (58.3)	19 (4.8)	9 (6–10)	16 (84.2)
	Zambia	2223	9 (5–14)	794 (35.7)	59 (2.7)	8 (4–12)	25 (42.4)
	Zimbabwe	2008	11 (7–17)	905 (45.1)	71 (3.5)	10 (7–16)	26 (36.6)
South-East Asia Region	India	372	9 (4–15)	104 (28.0)	67 (18.0)	9 (6–16)	33 (49.3)
	Myanmar	283	9 (6–13)	155 (54.8)	108 (38.2)	10 (6–13)	49 (45.4)
Western Pacific Region	Philippines	2641	12 (7–12)	1079 (40.9)	139 (5.3)	11 (7–12)	68 (48.9)
Region for the Americas	Brazil	557	16 (8–27)	86 (15.4)	154 (27.6)	20 (13–36)	37 (24.0)
Overall		14643	11 (7–16)	5374 (36.7)	840 (5.7)	11 (7–18)	382 (45.4)

Abbreviations: EIA, enzyme immunoassay; IQR, interquartile range; qPCR, quantitative polymerase chain reaction.

**Figure 2.**
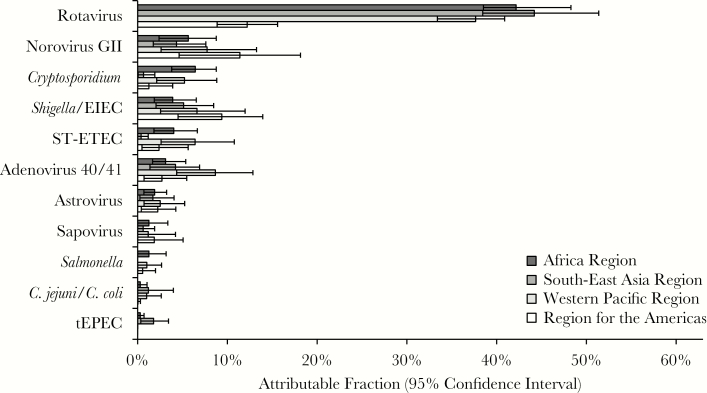
Pathogen-specific burdens of diarrhea using quantitative polymerase chain reaction by World Health Organization (WHO) region. Weighted attributable fraction (AF) is shown for each pathogen by WHO region. Pathogens are ordered by the overall AF. All pathogens for which the 95% confidence interval of the overall AF did not include 0 are shown. Abbreviations: EIEC, enteroinvasive *Escherichia coli*; ST-ETEC, heat-stable enterotoxin-producing *Escherichia coli*; tEPEC, typical enteropathogenic *Escherichia coli.*

**Figure 3.**
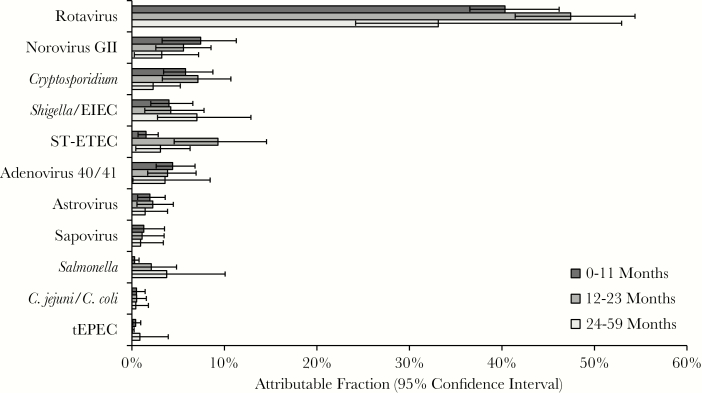
Pathogen-specific burdens of diarrhea using quantitative polymerase chain reaction by age group. Weighted attributable fraction (AF) is shown for each pathogen and age group. Pathogens are ordered by the overall AF. All pathogens for which the 95% confidence interval of the overall AF did not include 0 are shown. Abbreviations: EIEC, enteroinvasive *Escherichia coli*; ST-ETEC, heat-stable enterotoxin-producing *Escherichia coli*; tEPEC, typical enteropathogenic *Escherichia coli*.

To estimate pathogen-specific etiologies of diarrhea, we calculated AFs. We used inverse probability weighting to adjust for nonrandom sampling after inspection of the data suggested that the number of samples selected for qPCR testing was not proportional to the number of diarrheal episodes captured by surveillance at each site, and because not all sites met the goal of 50% EIA positivity and EIA-positive episodes appeared to be generally oversampled (Table 1). First, we calculated AFs both overall and for each region (Figure 1B and Figure 2). Overall, 70.8% (95% CI, 53.2%–92.8%) of diarrheal episodes were attributable to a pathogen. Rotavirus was the dominant etiology of diarrhea requiring hospitalization in all regions (weighted AF, 40.3% [95% CI, 37.6%–44.3%]) except the Americas, where monovalent RV was introduced in March 2006 in Brazil, the region’s single participating country for this study, and where the burden of rotavirus (AF, 12.2 [95% CI, 8.9–15.6]) and norovirus GII (AF, 11.4 [95% CI, 4.6–18.2]) was similar. In the overall estimates, norovirus GII (AF, 6.2 [95% CI, 2.8–9.2]), *Cryptosporidium* (AF, 5.8 [95% CI, 4.0–7.6]), *Shigella* (AF, 4.7 [95% CI, 2.8–6.9]), ST-ETEC (AF, 4.2 [95% CI, 2.0–6.1]), and adenovirus 40/41 (AF, 4.2 [95% CI, 2.9–5.5]) also demonstrated high AFs. *Cryptosporidium* and ST-ETEC AFs were particularly high in the Africa and Western Pacific regions, adenovirus 40/41 in the Western Pacific, and *Shigella*/EIEC in the Americas. Application of inverse probability weighting decreased the overall rotavirus AF from 48.0 (95% CI, 43.9–53.2) to 40.3 (95% CI, 37.6–44.3), which was consistent with the discrepancy in EIA positivity between samples selected for qPCR testing (45.4%) and all surveilled stools (36.7%). We then examined etiology in children by age (Figure 3). The rotavirus AF was highest in the first 2 years of life, ST-ETEC was prominent in the second year of life, and the *Shigella* AF increased with age.

Of the 12 countries from the Africa Region, 10 had introduced RV into the national immunization program by the end of 2014 (Table 2). Overall, about 40% of the children enrolled from these sites were age eligible to receive RV. Because individual vaccine histories were not available, we estimated the effectiveness of RV introduction by modeling the association between the country-specific age-eligibility status for each child aged 2–23 months in the Africa Region and the rotavirus EIA result. The effectiveness of RV introduction was 46.0% (95% CI, 34.5%–55.5%).

**Table 2.** Rotavirus Enzyme Immunoassay Result for Children 2–23 Months of Age Who Were Age Eligible and Age Ineligible to Receive at Least 1 Dose of Rotavirus Vaccine, for Africa Region Countries in Which Rotavirus Vaccine Was Introduced by 2014

Country	No.	Date of RV Introduction	RV Type	2013/2014 Coverage Estimates, %^a^	Age Eligible			Age Ineligible		
					No. (%)	Age (mo), Mean ± SD	Rotavirus EIA Positive, No. %	No. (%)	Age (mo), Mean ± SD	Rotavirus EIA Positive, No. (%)
Burkina Faso	694	November 2013	RotaTeq	9/91	155 (22.3)	6 ± 2.6	27 (17.4)	539 (77.7)	11.6 ± 4.3	292 (54.2)
Ghana	547	April 2012	Rotarix	87/98	468 (85.6)	10.4 ± 4.9	152 (32.5)	79 (14.4)	17.5 ± 3.6	28 (35.4)
The Gambia	186	August 2013	RotaTeq	90/92	40 (21.5)	7.5 ± 3.1	3 (7.5)	146 (78.5)	13.4 ± 5.3	41 (28.1)
Rwanda	1337	June 2012	RotaTeq	>99/98	1098 (82.1)	11 ± 4.3	211 (19.2)	239 (17.9)	16.2 ± 3.9	77 (32.2)
South Africa	1086	August 2009	Rotarix	89/94	1086 (100)	9.6 ± 5.2	314 (28.9)	0 (0.0)	NA	NA
Senegal	157	November 2014	Rotarix	NA/NA	0 (0.0)	NA	NA	157 (100.0)	9.7 ± 5.4	66 (42.0)
Sierra Leone	307	April 2014	Rotarix	NA/70	1 (0.3)	3.0 ± 0.0	0 (0.0)	306 (99.7)	9.4 ± 4.4	142 (46.4)
Togo	342	July 2014	Rotarix	NA/35	0 (0.0)	NA	NA	342 (100.0)	10.1 ± 4.6	203 (59.4)
Zambia	1816	December 2013	Rotarix	NA/73	374 (20.6)	6.1 ± 2.4	115 (30.7)	1442 (79.4)	10.8 ± 5.1	565 (39.2)
Zimbabwe	1712	May 2014	Rotarix	NA/82	80 (4.7)	5.2 ± 1.6	19 (23.8)	1632 (95.3)	11.1 ± 4.9	835 (51.2)
Overall	8184				3302 (40.3)	9.5 ± 4.8	841 (25.5)	4882 (59.7)	11.3 ± 5.1	2249 (46.1)

Abbreviations: EIA, enzyme immunoassay; NA, not applicable; RV, rotavirus vaccine; SD, standard deviation.

^a^Vaccine coverage estimates for a complete course (from the World Health Organization vaccine-preventable disease monitoring system, 2016 global summary; available at: http://apps.who.int/immunization_monitoring/globalsummary/timeseries/tscoveragerotac.html).

**Figure 4.**
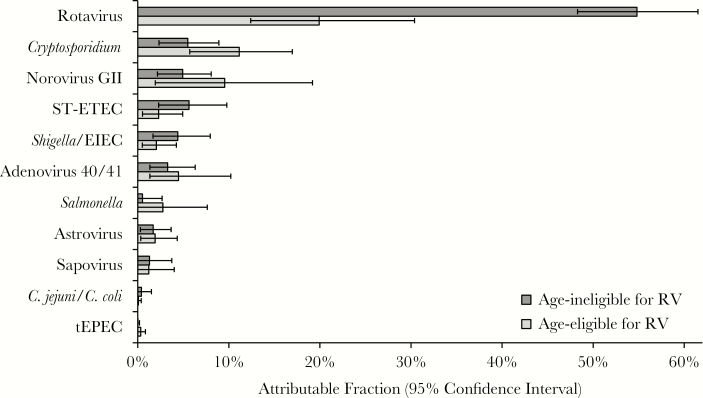
Impact of rotavirus vaccine (RV) introduction in the African Region for children aged 2–23 months in countries that introduced RV by 2014. Weighted attributable fraction (AF) is shown for each pathogen, stratified by age eligibility to receive RV. Children were considered age eligible if they were born no more than 2 months prior to the country’s month of RV introduction. Pathogens are ordered by the overall AF. All pathogens for which the 95% confidence interval of the overall AF did not include 0 are shown. Abbreviations: EIEC, enteroinvasive *Escherichia coli*; RV, rotavirus vaccine; ST-ETEC, heat-stable enterotoxin-producing *Escherichia coli*; tEPEC, typical enteropathogenic *Escherichia coli*.

We then calculated etiologies of diarrhea in age-eligible and age-ineligible children aged 2–23 months (Figure 4). Despite RV introduction, rotavirus remained the leading etiology of diarrhea requiring hospitalization in the first 2 years of life in the Africa Region by qPCR; however, the AF estimate was reduced from 54.8% (95% CI, 48.3%–61.5%) in age-ineligible children to 20.0% (95% CI, 12.4%–30.4%) in age-eligible children. After RV introduction, there appeared to be increases in the proportion attributable to *Cryptosporidium* and norovirus GII in age-eligible children; however, these were not statistically significant.

**Figure 5.**
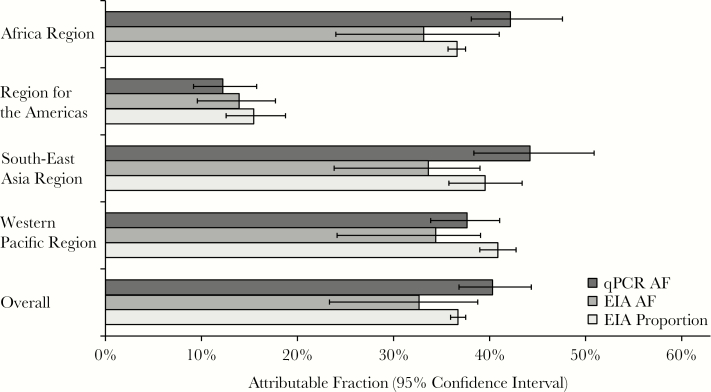
Rotavirus burden estimates by World Health Organization region using 3 distinct approaches. Rotavirus burden estimates are shown using (1) weighted attributable fractions (AFs) by quantitative polymerase chain reaction (qPCR); (2) weighted AFs by enzyme immunoassay (EIA); and (3) proportion of stools positive for rotavirus by EIA.

Finally, we compared rotavirus estimates between 3 approaches: (1) weighted AFs by qPCR (40.3%; 95% CI, 36.8%–44.3%); (2) weighted AFs by EIA (32.7% [95% CI, 23.3%–38.8%]); and (3) proportion of stools positive by EIA (36.7% [95% CI, 35.9%–37.5%]) (Figure 5). Consistent with our previous findings [4], the estimates were generally robust, with a slight increase in burden estimates by qPCR (9.8% increase vs EIA proportion positive, and 23.4% increase vs EIA AF).

## DISCUSSION

By applying molecular diagnostics, we leveraged this global surveillance network to identify the global etiologies of severe acute watery diarrhea in children <5 years of age. A strength of this work was the harmonized sample processing and testing of specimens from 16 countries, including many in Africa where limited epidemiologic data are available. Overall, rotavirus remained the dominant etiology despite the substantial impact of RV introduction in the Americas and Africa regions. We also found that 5 additional pathogens—norovirus GII, *Cryptosporidium*, *Shigella*/EIEC, ST-ETEC, and enteric adenovirus 40/41—were substantially and similarly important in these diverse settings.

Formal vaccine effectiveness studies are difficult to conduct in broad surveillance studies for which vaccine card confirmation of vaccination status is not available, but a comparison of RV age-eligible and age-ineligible children provided indirect evidence for a strong RV effect in Africa, with a 46% effectiveness of vaccine introduction. This estimate is, as expected, lower than estimates from clinical trials and vaccine effectiveness studies showing rotavirus vaccine effectiveness of approximately 50%–60% in African countries, for which individual-level vaccination histories were available [12–15]. Future testing will allow further assessment of vaccine effectiveness in diverse countries. More directly, by qPCR, we measured a >60% decline in rotavirus burden in children who were age eligible to receive RV. Moreover, the qPCR approach provides the context of the burden of nonrotavirus enteropathogens. This confirms that rotavirus remained the leading etiology of diarrhea requiring hospitalization in these countries even early after RV introduction, and highlights the importance of ongoing efforts to monitor the impact of vaccine introduction, maximize RV coverage, and continue research to understand and improve the low performance of RV in these settings [16]. It also shows the possibility for shifting etiologies post-RV, with particularly high rates of *Cryptosporidium* and norovirus GII in the Africa Region and norovirus GII in the Americas after RV introduction. Prospective surveillance will be important to confirm such changes.

The high burden of *Cryptosporidium*, *Shigella*/EIEC, ST-ETEC, and adenovirus 40/41 was not surprising, as these were important pathogens in the GEMS study [4, 5]. The high burden of *Shigella/*EIEC, although lower than that identified in the qPCR reanalysis of GEMS, was notable because the GRSN surveillance protocol excluded dysentery. In a survey of 5 countries included in the GRSN, dysentery represented 0–6% of all hospitalized diarrhea (unpublished data), but a significant proportion of this is likely attributable to *Shigella*/EIEC; thus, we may have underestimated the true burden of severe diarrhea attributable to this pathogen. It is likely that the *Shigella*/EIEC burden primarily reflects *Shigella flexneri* and *Shigella sonnei*, as has been found previously [4]. This finding reinforces the need for better diagnostics and treatments as well as enhanced vaccine development for *Shigella*. *Cryptosporidium* was particularly common across Africa, where it was found to have the second highest attributable burden of diarrhea in RV age-eligible children (after rotavirus), and where diarrheal mortality rates are highest. It has previously been identified as a high-risk pathogen in patients with human immunodeficiency virus and malnutrition [17, 18]. Thus, *Cryptosporidium* is also a highly important pathogen to address.

Norovirus was particularly important in the first 2 years of life, and was close to eclipsing rotavirus as the leading cause of diarrhea in the Americas, where rotavirus vaccination is established in the national immunization programs of most countries, including introduction in Brazil in 2006. This high prevalence of norovirus as an etiology of more severe diarrhea in children in low-resource countries was somewhat surprising, because its prevalence in moderate-to-severe diarrhea in the large GEMS study [4, 5] was relatively low. Differences in case definitions, particularly the exclusion of dysentery and a focus on hospitalized children, may be contributing to the different norovirus estimates. Additionally, this network reflects more recent epidemiology and includes post-RV settings. Altogether, these data suggest that norovirus is an important etiology of severe diarrhea in these settings.

We were surprised that *Campylobacter* was not implicated as a major pathogen in this study. This may be in part due to the exclusion of dysentery in the case definition as well as the high severity of illness. For example, *Campylobacter* was the pathogen most strongly associated with dysentery in infants but also the sole pathogen associated with a lower severity score in a multisite evaluation of the etiology of diarrhea in 8 low-income communities across the 3 continents [19]. Variation in pathogen burden between the specific sites included in these studies may also be an important factor.

We found a modest increase in rotavirus burden estimates when we used a qPCR-based AF methodology, which accounts for detection of rotavirus in controls per the GEMS models, vs an EIA-based AF methodology (23.4% underestimate vs qPCR AF) or the cross-sectional prevalence of rotavirus by EIA (9.8% underestimate vs qPCR AF), which does not account for detection in controls. This is in comparison to multifold increases in burden estimates for several other pathogens, including *Shigella*, ETEC, and adenovirus 40/41. Thus, cross-sectional prevalence, as has been used previously in the network [3], provides generally comparable burden estimates with that of qPCR, and we propose that either method may be used in the network in the future to obtain comparable estimates over time. For instance, in 2011–2012 the cross-sectional prevalence was 36% by EIA on 75353 tests [6], and here in 2013–2014 we observed a similar 37% cross-sectional prevalence and 40.3% attribution by qPCR.

Logistically, the technology worked well. We conducted a 1-week training session on-site, after which the laboratories ran all tests with excellent data quality (96% valid results). Compared with the multiple diagnostics needed to test for multiple enteropathogens with traditional methods, including culture and several EIAs, this approach greatly simplifies the protocol for broad enteropathogen testing and is cost effective [9].

This study had several limitations. First, several regions were represented by single surveillance sites. Because the network was established to track the burden of rotavirus diarrhea, it enrolled the subset of acute watery diarrhea to enrich the probability of rotavirus diarrhea. Specifically, dysentery was excluded, a small subset of hospitalized diarrhea but thought to be of clinical importance and for which antibiotic therapy is recommended [20]. Sample selection in each country for qPCR testing was not randomized, in particular due to oversampling of EIA-positive episodes. We calculated weighted estimates to adjust for this; however, better estimates of disease burden and effect of vaccination could be attained with prospective, randomized sampling. Some children in Brazil were not hospitalized, and as rotavirus is generally enriched in severe disease [4, 19], this may partially explain the relatively low burden of rotavirus in this region. Because control stools are not collected in the network, we used previously developed models from a reanalysis of the GEMS study to calculate AFs, which take into account both pathogen prevalence and the OR for diarrhea status. Our models derived only from watery diarrhea cases from GEMS, not dysentery, to better match the GRSN case definition. These imported models were developed for 20 pathogens and testing was performed for all but 1 (*Helicobacter pylori*) of these pathogens in this study, and 11 of the remaining 19 were identified as having a significant burden of diarrhea. Moreover, these models are based on the supposition that differential detection and pathogen quantity in cases vs healthy controls is an indication of etiology. This may have limitations for pathogens where pathogen quantity can be similar in symptomatic and asymptomatic infections [21].

In summary, in this retrospective analysis of stools from 16 countries, we document that rotavirus remained the dominant etiology of diarrhea requiring hospitalization, even in countries that had recently introduced RV, and despite sensitively testing for other enteropathogens. We would expect that the burden of rotavirus will continue to decline, but ongoing surveillance is needed to determine the residual burden of rotavirus and monitor the evolving etiologic distribution of diarrhea in these settings.

## Notes

***Acknowledgments***. We thank the study personnel from each participating surveillance site and the WHO regional reference laboratories for their many contributions to this study.

***Financial support.*** This work was supported by the Bill & Melinda Gates Foundation (grant number OPP1084328).

***Potential conflicts of interest.*** All authors: No reported conflicts of interest. All authors have submitted the ICMJE Form for Disclosure of Potential Conflicts of Interest. Conflicts that the editors consider relevant to the content of the manuscript have been disclosed.
